# Case Report: The Second Near-Infrared Window Indocyanine Green Angiography in Giant Mediastinal Tumor Resection

**DOI:** 10.3389/fsurg.2022.852372

**Published:** 2022-03-11

**Authors:** Jiahui Mi, Ganwei Liu, Liyang Lu, Feng Yang, Hui Zhao, Yun Li, Guanchao Jiang, Fan Yang, Zhenhua Hu, Jian Zhou

**Affiliations:** ^1^Department of Thoracic Surgery, Peking University People's Hospital, Beijing, China; ^2^Key Laboratory of Molecular Imaging of Chinese Academy of Sciences, Institute of Automation, Chinese Academy of Sciences, Beijing, China

**Keywords:** second near-infrared window, indocyanine green, angiography, mediastinal tumor, intraoperative imaging

## Abstract

Giant mediastinal tumors are often accompanied by the abundant blood supply and have an unclear border with adjacent vessels, making surgical resection difficult. Failure to distinguish the complex vessels during the operation often results in vascular injury or hemorrhage, which severely increases the operation time and perioperative risk. At present, surgeons can only determine the vessel's location and course by preoperative imaging and intraoperative exploration in visible light. Therefore, we report a case of a giant anterosuperior mediastinal tumor resection assisted by near-infrared (NIR) indocyanine green (ICG) angiography. Furthermore, we applied the second near-infrared window (NIR-II, 1,000–1,700 nm) to detect the fluorescence signals in the clinic for the first time. The NIR-II window is able to explore deeper tissues in centimeters and obtain higher resolution in millimeters than the traditional first near-infrared window (NIR-I, 700–900 nm). Finally, NIR-II ICG angiography shows the clear location and course of the vessels, which can help surgeons reduce unnecessary blood vessel injury and increase the safety of mediastinal tumor resection.

## Introduction

Resection of a giant mediastinal tumor is complicated in thoracic surgery because the thorax encompasses vital and complex vascular structures. At present, surgeons can only determine the vessel's location and course by preoperative imaging and intraoperative exploration in visible light. Although digital subtraction angiography can clearly show blood vessels, it is rarely used in open surgery because of its complicated operation and continuous X-ray exposure. Therefore, an appropriate real-time vascular imaging technique is needed to help surgeons identify blood vessels in mediastinal tumor resection.

Fluorescence imaging has shown several advantages with a high temporal and spatial resolution (1). First, near-infrared (NIR-I, 700–900 nm) indocyanine green (ICG) angiography has been applied in real-time vascular imaging in various surgical scenarios (2, 3). In addition, recent advances have shown that the second near-infrared window (NIR-II, 1,000–1,700 nm) can reduce light scattering by tissues and increase penetration depth, surpassing the NIR-I window's performance (1). To date, no clinical application has been reported for NIR-II ICG angiography.

Herein, we report a patient with a mediastinal tumor adjacent to the subclavian artery and vein who underwent a successful operation assisted by NIR-II ICG angiography for the first time.

## Case Report

**Figure 1:**
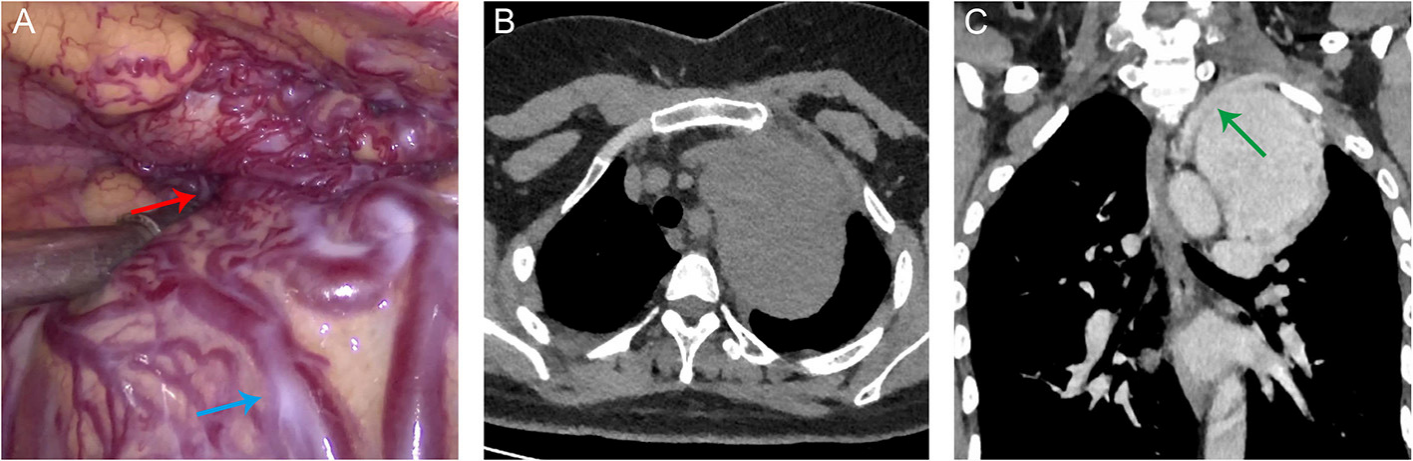
**(A)** Intraoperative photograph showing abundant tortuous blood vessels on the surface of the tumor (blue arrow) and an unclear border at the top of the chest (red arrow). **(B)** A CT scan revealed a giant anterosuperior mediastinal tumor (6.7 × 9.8 × 9.7 cm) near the left side of the aortic arch. **(C)** Contrast-enhanced CT scan showed that the tumor was surrounded by tortuous vessels and was poorly demarcated from the left subclavian artery (green arrow).

A 36-year-old woman presented with chest discomfort, wherein an anterosuperior mediastinal tumor had been diagnosed 3 weeks prior in the local hospital. Intraoperative exploration found abundant tortuous blood vessels on the surface of the tumor, and the surrounding tissue structure was unclear (Figure 1A), which easily caused uncontrollable bleeding. Therefore, the operation had to be terminated early.

Upon referral, she was admitted to our hospital for further treatment. Chest CT scan showed a giant anterosuperior mediastinal tumor (6.7 × 9.8 × 9.7 cm) near the left side of the aortic arch (Figure 1B). The tumor was surrounded by tortuous vessels on a contrast-enhanced CT scan and was poorly demarcated from the left subclavian artery (Figure 1C). A multidisciplinary discussion led to the decision to perform interventional embolization to decrease the tumor volume and the blood supply to the tumor in preparation for the subsequent surgical resection.

A contrast-enhanced CT scan was reviewed 7 days after interventional embolization, and the volume and blood supply of the mediastinal tumor were slightly reduced compared with before. After preoperative inspection, no contraindication was found in this patient, and we decided to perform mediastinal tumor resection.

**Figure 2:**
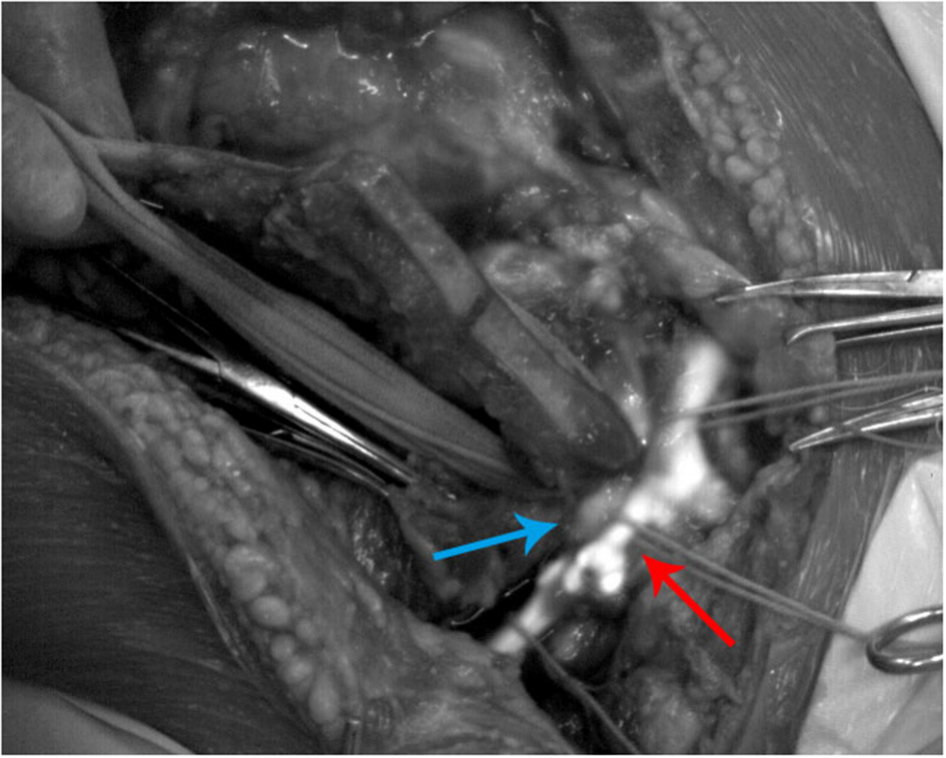
Second near-infrared (NIR-II) indocyanine green (ICG) angiography of the left subclavian vein (red arrow) and the left subclavian artery (blue arrow) during the operation.

The patient was anesthetized in the supine position. We split the sternum in the middle, disconnected the sternoclavicular joint, and removed half of the left clavicle to sufficiently expose the tumor. Intraoperative exploration found that the upper pole of the tumor was located at the entrance of the thorax and was closely adhered to the surrounding tissues. To distinguish the subclavian artery and vein from the surrounding tissues, the upper pole of the tumor was imaged in real-time by a NIR-II imaging system. The NIR-II imaging system contained a camera (Cheetah 640, Xenics, Belgium) using an InGaAs detector with 640 x 512-pixel resolution, a lens specially designed for high-performance shortwave infrared imaging (SWIRON 2.8/50, Schneider Kreuznach, Germany), and a spectral filter (FELH1000, Thorlabs, USA). A 792-nm laser (Bosheng Optoelectronics Co. Ltd., China) beam was coupled to a collimator and expanded by a lens to provide uniform illumination on the upper pole of the tumor. Then, 25 mg (5 mg/ml) ICG (Dandong Yichuang Pharmaceutical Co. Ltd., China) was injected into the patient's left radial vein in 10 s. The left subclavian vein and the left subclavian artery showed obvious fluorescence signals (Figure 2) and displayed a clear boundary of 3 and 17 s after the initial injection of ICG, respectively (Supplementary Video 1). With the help of NIR-II ICG angiography, we could recognize the deeper position of the subclavian vein and artery and avoid vascular damage during the operation. In addition, the clear blood vessel borders visualized by NIR-II ICG angiography ensured that the subclavian vein and artery were dissected free of all surrounding tissue without any vascular injury.

No allergic symptoms occurred after the ICG injection, and no severe nausea or vomiting occurred after the operation. Histologic examination of the tumor revealed a solitary fibrous tumor, and this patient's postoperative course was uneventful at follow-up.

## Discussion

Real-time NIR-I fluorescence imaging *in vivo* has received significant recognition as a powerful technique. It has been used in thoracic surgery, such as lung nodule identification (4). However, there is growing interest in NIR-II fluorescence imaging research due to reducing light scattering by tissues and increasing penetration depth compared to NIR-I fluorescence imaging (1). In addition, ICG as a non-targeting fluorescence agent has been approved for clinical use by the US Food and Drug Administration (FDA) for more than six decades and has been proven to be suitable for NIR-II imaging (5). Although animal experiments have proven that NIR-II ICG angiography has advantages over NIR-I ICG angiography (6), NIR-II ICG angiography has not yet been applied in clinical practice.

In our patient, the subclavian vein began to show fluorescence signals 3 s after ICG was injected into the radial vein. The subclavian artery began to show fluorescence signals 17 s after the blood combined with ICG passed through the pulmonary circulation and flowed into the subclavian artery. Therefore, intravenous injection of ICG from the upper limb had quick feedback for vascular fluorescence imaging in the mediastinum. In addition, NIR-II fluorescence imaging can show a more apparent vascular boundary by the characteristics of diminishing tissue autofluorescence, reducing photon scattering, and exhibiting lower levels of photon absorption at longer wavelengths (7), which could help surgeons identify complex vascular structures and reduce the risk of vascular injury.

The video demonstrated that the fluorescence signal intensity in the subclavian vein was not homogeneous at the beginning. Fluorescence imaging of the subclavian vein even showed apparent blood flow turbulence and vortex in the vascular bifurcation. This phenomenon is due to ICG being injected only into the radial vein. In other veins, such as the superior scapular vein, internal jugular vein, and external jugular vein, blood without ICG flowed into the subclavian vein together. The precise blood flow imaging proved that NIR-II ICG angiography has a relatively high resolution, consistent with previous research (8). Therefore, if the blood vessels had to be reconstructed due to severe injury or tumor invasion during the operation, NIR-II ICG angiography could also monitor the vessels' blood flow and assist surgeons in judging whether the reconstructed vessels were unobstructed in real-time. Compared with traditional X-ray vascular imaging, NIR-II ICG angiography can show blood flow continuously without considering radiation damage to patients and medical personnel.

## Conclusion

Our case is the first reported clinical application of NIR-II ICG angiography in mediastinal tumor surgery and showed that NIR-II ICG angiography, as a new clinical imaging technique, is a safe, convenient, and efficient real-time method to help surgeons deal with vital vessels in mediastinal tumor operations in the future.

## Data Availability Statement

The original contributions presented in the study are included in the article/Supplementary Material, further inquiries can be directed to the corresponding authors.

## Ethics Statement

Written informed consent was obtained from the individual(s) for the publication of any potentially identifiable images or data included in this article.

## Author Contributions

JM reviewed the literature and drafted the manuscript. GL and LL collected the analyzed the data. FeY, HZ, and YL provided technical support. GJ and FaY contributed to the conception and design of the study. ZH and JZ reviewed the manuscript. All authors issued approval of the final version.

## Funding

This study was supported by the National Natural Science Foundation of China (Nos. 92059203 and 82003316).

## Conflict of Interest

The authors declare that the research was conducted in the absence of any commercial or financial relationships that could be construed as a potential conflict of interest.

## Publisher's Note

All claims expressed in this article are solely those of the authors and do not necessarily represent those of their affiliated organizations, or those of the publisher, the editors and the reviewers. Any product that may be evaluated in this article, or claim that may be made by its manufacturer, is not guaranteed or endorsed by the publisher.

## Supplementary Material

Supplementary Video 1 The indocyanine green second near-infrared real-time angiography for subclavian vein and artery.
